# Dual-Layer Spectral CT with Electron Density in Bone Marrow Edema Diagnosis: A Valid Alternative to MRI?

**DOI:** 10.3390/jcm14155319

**Published:** 2025-07-28

**Authors:** Filippo Piacentino, Federico Fontana, Cecilia Beltramini, Andrea Coppola, Daniele Mesiano, Gloria Venturini, Chiara Recaldini, Roberto Minici, Anna Maria Ierardi, Velio Ascenti, Simone Barbera, Fabio D’Angelo, Domenico Laganà, Gianpaolo Carrafiello, Giorgio Ascenti, Massimo Venturini

**Affiliations:** 1 Department of Diagnostic and Interventional Radiology, Circolo Hospital and Macchi Foundation, Insubria University, 21100 Varese, Italy; filippo.piacentino@asst-settelaghi.it (F.P.); federico.fontana@uninsubria.it (F.F.); cbeltramini@studenti.uninsubria.it (C.B.); andrea.coppola@asst-settelaghi.it (A.C.); dmesiano@studenti.uninsubria.it (D.M.); gloriaventurinivarese@gmail.com (G.V.); chiara.recaldini@asst-settelaghi.it (C.R.); 2 Department of Medicine and Technological Innovation (DiMIT), Insubria University, 21100 Varese, Italy; 3 Radiology Unit, Department of Experimental and Clinical Medicine, University Hospital Mater Domini, Magna Graecia University of Catanzaro, 88100 Catanzaro, Italy; roberto.minici@unicz.it (R.M.); domenico.lagana@unicz.it (D.L.); 4 Interventional Radiology Unit, Department of Radiology, Foundation IRCCS Ca’ Granda-Ospedale Maggiore Policlinico, 20126 Milan, Italy; annamaria.ierardi@policlinico.mi.it (A.M.I.); velio.ascenti@policlinico.mi.it (V.A.); gianpaolo.carrafiello@unimi.it (G.C.); 5 Diagnostic and Interventional Radiology Unit, BIOMORF Department, University Hospital “Policlinico G. Martino”, 98124 Messina, Italy; simone.barbera@studenti.unime.it (S.B.); giorgio.ascenti@unime.it (G.A.); 6 Department of Medicine and Surgery, Insubria University, 21100 Varese, Italy; fabio.dangelo@uninsubria.it; 7 Orthopedic Surgery Unit, ASST Sette Laghi, 21100 Varese, Italy

**Keywords:** MRI 1, Dual-Layer Spectral CT 2, bone marrow edema 3

## Abstract

**Background/Objectives**: Although MRI with fat-suppression sequences is the gold standard for diagnosis of bone marrow edema (BME), Dual-Layer Spectral CT (DL-SCT) with electron density (ED) provides a viable alternative, particularly in situations where an MRI is not accessible. Using MRI as the reference standard, this study analyzed how DL-SCT with ED reconstructions may be a valid alternative in the detection of BME. **Methods**: This retrospective study included 28 patients with a suspected diagnosis of BME via MRI conducted between March and September 2024. Patients underwent DL-SCT using ED reconstructions obtained through IntelliSpace software v. 12.1. Images were evaluated by two experienced radiologists and one young radiologist in a blinded way, giving a grade from 0 to 3 to classify BME (0 absence; 1 mild; 2 moderate; 3 severe). To reduce the recall bias effect, the order of image evaluations was set differently for each reader. *p*-Values were considered significant when <0.05. Fleiss’ Kappa was used to assess inter-rater reliability: agreement was considered poor for k < 0; slight for k 0.01–0.20; fair for 0.21–0.40; moderate for 0.41–0.60; substantial for 0.61–0.80; and almost perfect for 0.81–1.00. **Results**: All the readers detected the presence or absence of BME using DL-SCT. Inter-rater reliability for grade 0 resulted in 1 (*p*-value < 0.001); for grade 1: 0.21 (*p*-value < 0.001); for grade 2: 0.197 (*p*-value < 0.001); and for grade 3: 0.515 (*p*-value < 0.001). **Conclusions**: ED reconstructions allowed the identification of BME presence or absence in all analyzed cases, thus suggesting DL-SCT as a potentially effective method for its detection.

## 1. Introduction

Bone marrow edema (BME) is a condition characterized by fluid accumulation within the bone associated with trauma, inflammation, neoplasms, or degenerative diseases. Accurate and timely diagnosis of BME is crucial for patient treatment [1].

Currently, the preferred method for assessing BME remains MRI with fat-suppression sequences [2,3,4,5]. However, the possibility of performing an emergency MRI is often limited to cases involving bone marrow involvement, which frequently results in delayed diagnosis [6]. Furthermore, MRI is expensive, time-consuming, and less accessible, and some patients may have contraindications [7,8]. Moreover, MRI requires significantly more time compared with a routine CT scan, often leading to patient discomfort and a higher likelihood of motion artifacts [9]. On the other hand, MRI offers the notable advantage of being a radiation-free imaging modality, which, conversely, represents one of the major issues to be taken into account when using CT scans.

The potential ability to identify BME using CT remains a radiological mirage that could transform the diagnostic approach to bone pathology, both in trauma and oncology.

CT currently has the technical limitation of being unable to detect BME due to the minimal changes in tissue density, which would result in negligible differences in Hounsfield units [10]. Additionally, the presence of calcified bone trabeculae implies a high atomic number within the voxel, making it impossible to distinguish between BME and blood in the tissue. Due to these technical limitations, CT remains solely useful for evaluating the cortical bone [11,12].

Recently, the use of dual-energy CT has been explored and demonstrated how dual-energy technology offers the capability to identify BME. In the literature, numerous studies have proposed various data processing methods for X-ray imaging to highlight BME [13,14].

Dual-energy CT, particularly with virtual non-calcium images, has shown excellent diagnostic accuracy for detecting BME, achieving a sensitivity of 85% and a specificity of 97% [15]. While both qualitative and quantitative assessments of dual-energy CT findings demonstrated outstanding diagnostic performance, qualitative assessment outperformed quantitative methods [16]. Several studies have already highlighted the effectiveness of spectral CT in identifying BME, particularly in vertebral fractures, across both the appendicular skeleton and the axial skeleton [17,18].

To our knowledge in the literature, excluding Ca suppression, no other processing tools are available to highlight BME. Using Dual-Layer Spectral CT (DL-SCT), a variant of the conventional dual-energy technology developed, the electron density (ED) mapping system has been identified as a potential tool for detecting variations in electron concentration within the voxel. This variation seems to allow edema detection [19,20,21], and, consequently, ED image reconstruction has shown a potential improvement in the detection and characterization of BME with plain CT. Our study was based on the hypothesis that electron density (ED) reconstructions obtained through Dual-Layer Spectral CT (DL-SCT) may serve as a reliable and effective tool for detecting bone marrow edema (BME). Given the lack of comparative data between DL-SCT ED mapping and MRI, currently considered the gold standard, we aimed to preliminarily verify whether ED reconstructions correlate with BME findings observed on MRI T2 FAT-SAT sequences. Our analysis intended to explore both the diagnostic potential and clinical applicability of this approach.

## 2. Materials and Methods

### 2.1. Study Design

In order to compare the two imaging modalities in assessing the degree of BME, we conducted a retrospective observational study involving patients with a suspected diagnosis of BME who underwent both MRI and DL-SCT of the corresponding anatomical region. Subsequently, the acquired images were independently reviewed in a blinded fashion by three radiologists, who were asked to assign a numerical score reflecting the severity of BME. The scores were then categorized and analyzed to derive statistical data and assess the concordance between the two techniques.

### 2.2. Sample Selection and Analysis

All patients enrolled in the study provided informed consent, including for the publication of anonymous data. Through a filter-based research set on the RIS available at our institution, we selected 36 patients who underwent an MRI scan with a suspected diagnosis of BME between March and September 2024: the presumed leading cause of edema and the affected anatomical district have not been considered as discriminants in the selection of the sample. The filters used for the preliminary informatics research are specified in the Supplementary Materials S1.

Out of 36 patients who satisfied our pre-established research criteria, we selected those who underwent a comprehensive spectral CT of the edematous region, performed before or immediately after the MRI (32 patients), with a maximum interval of 7 days between the two modalities (mean interval: 4.2 days). Furthermore, to have a homogeneous sample, patients with MRI radiological investigations performed in other institutions were excluded (4 patients); this narrowed the population down to 28 patients. Of these patients, 18 presented with BME on MRI examination, and 10 were negative for BME diagnosis. Inclusion and exclusion criteria are explicitly detailed in Table 1.

All of the population was analyzed based on sex, age, and comorbidities; the positive sample was classified based on BME etiology and involved region, as reported in Table 2, Table 3 and Table 4.

### 2.3. MRI Acquisition

All patients underwent a non-contrast MRI with a 1.5T magnetic resonance prior to placement of a dedicated surface coil for the anatomical region under investigation. For the study of BME using a Siemens MRI scanner (SOLA; Siemens Healthineers, Erlangen, Germany), the used sequences without contrast were STIR (Short Tau Inversion Recovery) and PD Fat Sat (Proton Density Fat Saturation). Below are the parameters for these sequences.

STIR Sequence (Short Tau Inversion Recovery) TR (Repetition Time): 3000–4500 ms; TE (Echo Time): 30–60 ms; TI (Inversion Time): ~150–180 ms (optimized for fat suppression); FOV (Field of View): 280–450 mm (depending on the region); slice thickness: 5 mm; slice gap: 10–20% of the slice thickness; matrix: 256 × 256 or higher for better resolution; acquisition time: ~2–5 min.

PD Fat Sat sequence (Proton Density Fat Saturation) was used for enhancing contrast between BME and surrounding tissues with selective fat suppression. Plane: sagittal, coronal, or axial; TR (Repetition Time): 2500–4000 ms; TE (Echo Time): 30–40 ms (for good image quality); FOV (Field of View): 280–450 mm; slice thickness: 3–5 mm; slice gap: 10–20% of the slice thickness; matrix: 256 × 256 or higher; fat suppression: chemical fat saturation (Fat Sat); acquisition time: ~3–4 min.

### 2.4. DL-SCT Acquisition

All patients underwent a non-contrast DL-SCT (IQon Spectral CT Philips, Amsterdam, The Netherlands), a 128-slice MDCT single-source dual-layer detector spectral scanner. All the images were taken with prefixed protocols, targeted to the region of interest, and with relevance to the clinical question, with spectral row data SBI (spectral-based images) acquisition. The CT scan parameters are specified in the Supplementary Materials S2.

### 2.5. DL-SCT Reconstruction Using ED Tool

The images acquired using DL-SCT were subsequently loaded and processed using Philips IntelliSpace Portal v. 12.1 (Philips, Amsterdam, The Netherlands), an advanced visualization and analysis platform that enables image reconstruction from raw spectral data through a variety of dedicated tools. For the purpose of this study, the ED map reconstruction was used, an advanced tool for tissue characterization that provides a quantitative estimation of the ED for each voxel. The values are expressed as a percentage relative to the electron density of water, which is defined as 100 when corresponding to 3.34 × 10^29^ electrons/m^3^. The resulting map is displayed in a colorimetric format, with a color code that varies according to the level of ED, allowing for quantification of the number of electrons per unit volume within the tissues. This type of analysis enables the detection of subtle variations in tissue composition, improving the differentiation between soft tissues, bone, calcifications, and other structures. No conversion from Hounsfield units is required: the ED information is intrinsic to the acquired spectral raw data, and no modification of the acquisition protocol is necessary.

### 2.6. Grading System

To effectively compare the two different imaging methods under analysis, we decided to assign a numerical value representing the degree of BME to each image obtained through MRI and Spectral CT imaging. Consequently, a point-based grading system was defined as illustrated in Table 4: absence of BME (0 points = grade 0); mild BME (1 point = grade 1); moderate BME (2 points = grade 2); severe BME (3 points = grade 3) (Table 5).

After defining the grading scale, three radiologists with experience in musculoskeletal imaging (two board-certified radiologists with 10 years of experience and one radiology resident with 2 years of experience) who were blinded to both the imaging reports and the clinical history were asked to review the scans and to give a score of BME to long TR sequences with fat signal suppression in MRI and the spectral reconstructions using ED for CT, previously obtained through IntelliSpace Software.

To reduce the effect of recall bias for each specific imaging set, the order of imaging assessments was set randomly and differently for each reader: the revision was performed independently and using a PACS viewer solution (Agfa Impax, Agfa HealthCare, Mortsel, Belgium).

### 2.7. Data Analysis and Statistics

All the statistical analyses were conducted with IBM SPSS Statistics Software, version 25.0, using Fleiss’ Kappa test to assess inter-rater reliability. Agreement was considered poor for k < 0; slight for k 0.01–0.20; fair for 0.21–0.40; moderate for 0.41–0.60; substantial for 0.61–0.80; and almost perfect for 0.81–1.00, as shown in Table 6; *p*-values were considered significant when <0.05 (Table 5).

## 3. Results

The results from each reader’s judgment of the BME grade have been sorted in a data matrix, as shown in Table 7.

All of the readers expressed a uniform judgement about the presence/absence of BME (grade from 0 to 3) in all sets of CT imaging obtained with ED reconstruction from spectral row data. 

Out of 28 patients, in ten cases, all of the readers expressed a judgment of absence of BME, attributing a grade of 0.

Inter-rater reliability for edema grade 0 resulted in 1 (*p*-value < 0.001), grade 1 resulted in 0.21 (*p*-value 0.001), grade 2 resulted in 0.197 (*p*-value < 0.001), and grade 3 resulted in 0.515 (*p*-value < 0.001), thus showing a significant agreement between all the readers (Table 8).

Intra-rater reliability calculated on the positive cases alone (18 patients) was higher in the third reader than in the board-certified radiologists. As shown in Table 8, the statistical discrepancy tends to manifest more prominently with grades 2 and 3, suggesting a greater variability in evaluation even within the same reviewer (Table 9).

**Figure 1:**
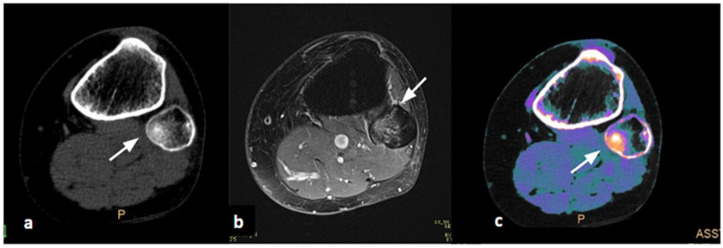
Female, 61 years old, sprain injury. (**a**) Axial CT image, baseline acquisition of the knee, does not show cortical alterations (white arrow). (**b**) Axial MRI acquisition demonstrates edema of the fibular head in PD/T2 FAT-SAT, indicating acute post-traumatic interstitial hemorrhage (white arrow). (**c**) Axial Dual-Layer Spectral CT (DL-SCT) image, post-processed with the IntelliSpace software, shows the presence of edema through an increase in electron density (ED) visible with orange coloring (white arrow). Color bar legend. White: normal cortical bone; orange: intermediate to high ED; purple: low ED; blue: very low ED, consistent with normal bone marrow composition rich in fat.

**Figure 2:**
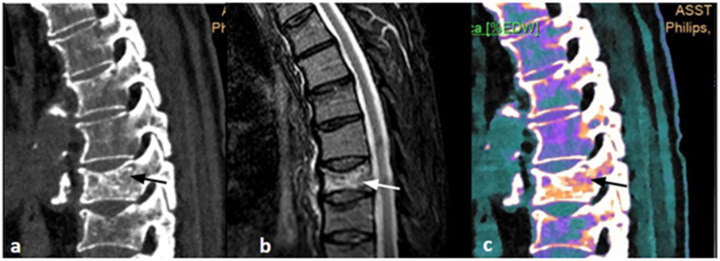
(**a**) Sagittal CT image, baseline acquisition of the thoracic spine, showing only the osteodense aspect of the vertebral body of D8 (black arrow). (**b**) Sagittal MRI acquisition with STIR sequence, clearly showing the hyperelectronicity of the D8 vertebral body, indicative of interstitial edema (white arrow) and an acute process. (**c**) Sagittal DL-SCT image, post-processed with IntelliSpace software, showing an increase in ED at the level of D8 vertebral body (orange color, black arrow), indicative of spongy edema. Color bar legend. White: normal cortical bone; orange: intermediate to high ED; purple: low ED; blue: very low ED, consistent with normal bone marrow composition rich in fat.

**Figure 3:**
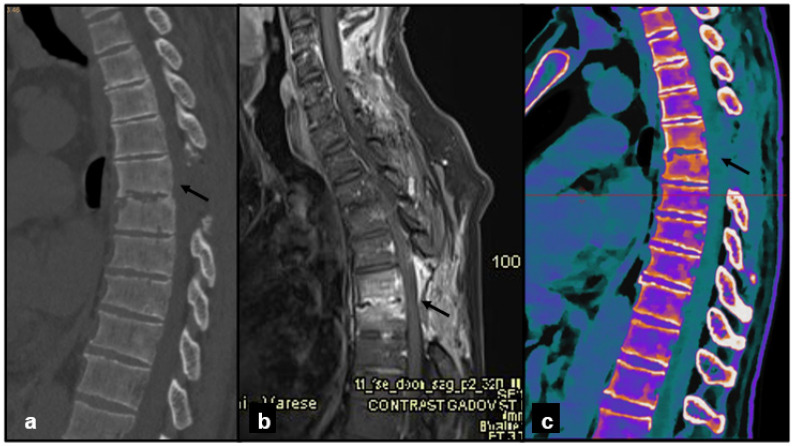
A 55-year-old male, work-related trauma. (**a**) Sagittal CT image, baseline acquisition of the thoracic spine shows intervertebral disc alteration with cortical irregularities of the opposing endplates (black arrow). (**b**) Sagittal MRI acquisition with PD/T2 FAT-SAT demonstrates intraspongious edema of the D5 and D6 vertebral bodies, indicating acute interstitial hemorrhage (black arrow). (**c**) Sagittal DL-SCT image, post-processed with IntelliSpace system, highlights the presence of edema through increased electron density, visualized in orange coloring (black arrow). Color bar legend. White: normal cortical bone; orange: intermediate to high ED; purple: low ED; blue: very low ED, consistent with normal bone marrow composition rich in fat.

**Figure 4:**
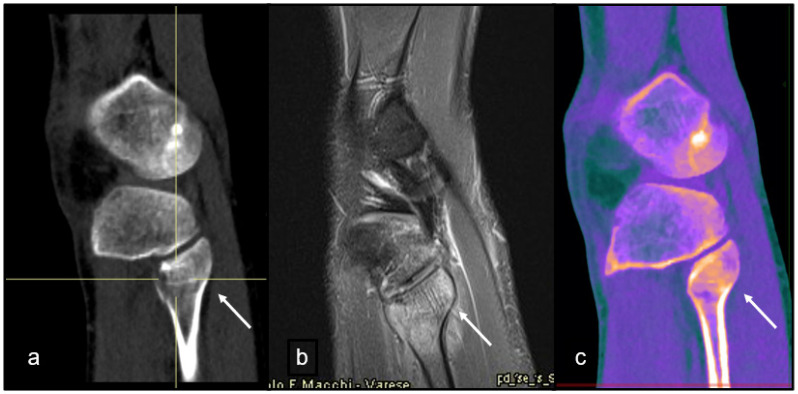
A 25-year-old male, sprain injury. (**a**) Coronal CT image, baseline acquisition of the knee shows no cortical alterations. (**b**) Sagittal MRI acquisition in PD/T2 FAT-SAT demonstrates edema of the fibular head, indicating acute post-traumatic interstitial hemorrhage (white arrow). (**c**) Sagittal DL-SCT image, post-processed with the IntelliSpace software, highlights the presence of edema through increased electron density, visualized in orange coloring (white arrow). Color bar legend. White: normal cortical bone; orange: intermediate to high ED; purple: low ED; blue: very low ED, consistent with normal bone marrow composition rich in fat.

At the end of the study, image collages were created to compare CT, MRI, and DL-SCT at the point of BME detection (Figure 1, Figure 2, Figure 3 and Figure 4).

## 4. Discussion

Using MRI as the reference standard, this study had the primary aim to evaluate the reliability of DL-SCT with ED reconstructions in the detection of BME.

Through a retrospective analysis, it was possible to collect and review some significant data that led us to explore the potential diagnostic capabilities of DL-SCT.

The results highlighted how DL-SCT was able to identify BME with a high negative predictive value in negative cases and a good intra-reader agreement for mild severity BME in positive patients, for which all the reviewers expressed uniform judgements.

This possibility has been known since the end of 2020, when the adoption of spectral CT with dual-layer technology was implemented in the clinical routine thanks to the introduction of two overlapping detectors, each capable of absorbing a different spectrum of energy [22].

The DL-SCT technique allows for distinguishing different materials through specific material density measurements based on the different properties of X-ray absorption, a step forward compared with the measurements in Hounsfield units [23]. Furthermore, compared to dual-energy CT, no pre-established acquisition protocols need to be selected to collect and analyze spectral data.

Material decomposition tools in dual-source and dual-energy CT allow the reconstruction of virtual images without calcium signal (CaSupp), which is selectively subtracted from the acquisition set of images: this technology is very useful in detecting pathological bone abnormalities such as BME, which would otherwise be missed at diagnosis by the presence of calcium itself.

Until recently, most of the studies found in the literature regarding the diagnosis of vertebral BME with virtual non-calcium reconstruction were conducted using first and second-generation dual-source dual-energy CTs. Lately, Petritsch et al. [24] have demonstrated that CaSupp maps from third-generation spectral CT offer better diagnostic performance thanks to a higher image quality. According to their study, the accuracy value in detecting acute bone fracture is 0.94, which is very similar to our results. Although direct comparisons between electron density-based BME detection and other DECT techniques such as CaSupp or VNCa are not available in the literature, our results suggest a diagnostic accuracy that closely aligns with those reported.

Another study conducted by Kaup et al. [25] compared second- and third-generation dual-source dual-energy CTs from different companies operating with various acquisition modalities, and, as a result, the diagnostic accuracy was revealed to be inferior to that of Petritsch’s study and ours, suggesting that BME detection could be sensitive to both technical acquisition factors and radiologists’ personal expertise in interpreting material-specific density maps [26].

According to Schwaiger et al. [27], who analyzed the application of three-material decomposition with DL-SCT in the diagnosis of edema, our study revealed that as the severity of BME increases, particularly in grades 2 and 3, an increase in inter-observer agreement has been observed.

This finding suggests that mild BME is inherently more challenging to identify consistently on DL-SCT, most likely due to its subtle imaging features and the intrinsic limitations of CT, being based on radiographic transparency, which further complicate the detection of soft tissue changes. Importantly, this may also account for the observed increase in intra-rater reliability with higher edema grades. In contrast, more advanced stages of BME tend to display clearer morphological characteristics, which may facilitate a greater level of diagnostic consistency among and within readers; it is probable that a wider sample could strengthen the statistical validity of these results.

In our preliminary experience, the intra-rater reliability was revealed to be higher in less experienced observers than in board-certified radiologists, implying that individual experience has a strong influence on evaluation and meanwhile can create learning opportunities and specific diagnostic improvements. 

Consequently, it is of paramount importance to highlight the impact that continuous and dedicated training has on diagnosis quality, especially in the complex field of musculoskeletal imaging. 

Another factor to consider is that ED, differently from CaSupp, is not directly affected by the age of the population. That is why we included a heterogeneous sample entirely comparable to the patients selected in previous studies.

Regarding CaSupp images, the fatty component of bone marrow, which is usually higher in the elderly than in younger individuals, can facilitate the differential diagnosis between BME and highly fatty bone marrow. On the other hand, young patients have a high percentage of red bone marrow, thus leading to a more challenging identification of edema [28].

This limitation has been easily overcome with ED because no significant difficulty in the detection of edema of the younger population has been reported. 

Future studies will be required to better understand how relevant the composition of bone marrow is in the different age groups for the diagnosis of BME and how it can influence the interpretation of spectral CT images. 

Despite the results still confirm MRI the gold standard in the diagnosis of BME, much more reliable than CT imaging, these recent results have clearly revealed that DL-SCT with ED maps has the potential to provide valid diagnostic information: this encouraging finding should serve as an impetus to optimize and validate the broader application of this new technology, not only as a diagnostic tool but also as a reliable guide for interventional procedures, such as bone infiltrations and tumor bone ablation, to understand the immediately post-ablation results [29]. Furthermore, the correlation between spectral imaging and PET results could improve the diagnostic accuracy and the planning of procedures such as lung biopsies [30].

One of the strengths of our study is that, unlike the previously cited ones, it did not focus solely on traumatic conditions in the elderly but included various pathological situations that can cause BME. On the other hand, as a limitation, our sample was very heterogeneous, and we did not consider metabolic disorders different from osteoporosis or malignancy. 

A future evaluation should also be able to differentiate between benign and malignant fractures, providing strong support in the complex clinical context of osteoporotic patients, where old and acute vertebral fractures coexist and guide therapeutic decisions.

Furthermore, the population sampled in this retrospective study was relatively small due to the very recent application of DL-SCT with ED in our center. Moving forward, future studies should aim to incorporate radiation dose considerations more explicitly, taking into account patient age, cumulative exposure, and risk–benefit assessment tailored to clinical indication. Dose optimization strategies, low-dose protocols, and age-specific diagnostic pathways may help refine the application of this technique in more vulnerable populations, including pediatric and young adult patients.

Based on the current literature, bone marrow edema remains clearly detectable on imaging for at least 10 days and often up to 30–40 days following the acute event. To build a reasonable comparison between CT and MR, we only included the patients who underwent both imaging modalities within 7 days [31].

Finally, this study solely analyzed the diagnostic performance of IQon Spectral CT, dual-energy Dual-Layer Spectral CT by Philips, using a one-material decomposition algorithm to obtain ED maps.

New roots of diagnostic imaging would be open with a further implementation of this technology; as a consequence, clinical practice would be optimized, providing a global improvement of patient care.

## 5. Conclusions

Dual-Layer Spectral CT-based evaluation of bone fractures with electron density reconstruction could improve the accuracy in both detection and classification of vertebral bone fractures, providing a promising tool to distinguish acute and chronic events without the need for MRI.

This study has underlined that most of the limiting factors of MRI application in acute settings, such as long acquisition time, high costs, and incompatible devices, would be easily overcome by the wider use of dual-layer technology, a high-potential instrument for daily clinical practice.

However, studies with larger sample sizes and greater statistical robustness will be necessary to confirm these preliminary findings.

## Figures and Tables

**Table 1:** Inclusion and exclusion criteria.

Inclusion Criteria
Patients with a suspected diagnosis of BME
Patients who underwent MRI and DL-SCT examinations at our institution
Patients with both MRI and spectral CT covering the region affected by edema
Interval between MRI and Spectral CT ≤ 7 days (mean: 4.2 days)
Patients with either a positive or negative bone marrow edema (BME) diagnosis on MRI
**Exclusion Criteria**
Patients who underwent MRI or DL-SCT at other outside institutions
Patients with an interval of >7 days between MRI and spectral CT

**Table 2:** Sex and age distribution in the sample.

SEX		AGE			
M	F	20–45	45–70	70–90	>90
18	10	8	14	4	2

**Table 3:** Comorbidity distribution in the sample.

COMORBIDITY				
HYPERTENSION	HYPERLIPIDEMIA	DIABETES T2	OSTEOPENIA	OSTEOPOROSIS
12	7	4	8	6

**Table 4:** Edema etiology and involved region distribution in the positive sample.

EDEMA ETIOLOGY		INVOLVED REGION			
TRAUMA	DEGENERATIVE DISEASE	PELVIC GIRDLE	KNEE	ANKLE	VERTEBRAL BODY
10	8	4	5	2	7

**Table 5:** Edema grading system.

	GRADE
ABSENCE	0
MILD	1
MODERATE	2
SEVERE	3

**Table 6:** Agreement level setting.

VALUE	AGREEMENT LEVEL
<0	POOR AGREEMENT
0.01–0.20	SLIGHT AGREEMENT
0.21–0.40	FAIR AGREEMENT
0.41–0.60	MODERATE AGREEMENT
0.61–0.80	SUBSTANTIAL AGREEMENT
0.81–1.00	ALMOST PERFECT AGREEMENT

**Table 7:** Results data matrix.

	MRI			CT		
	R1	R2	R3	R1	R2	R3
**1**	3	3	3	2	2	1
**2**	3	3	2	3	3	3
**3**	3	3	3	3	3	3
**4**	0	0	0	0	0	0
**5**	2	2	2	1	1	1
**6**	0	0	0	0	0	0
**7**	0	0	0	0	0	0
**8**	3	3	3	3	2	2
**9**	3	3	3	3	3	3
**10**	3	3	3	2	2	2
**11**	0	0	0	0	0	0
**12**	2	3	3	2	2	2
**13**	1	2	1	3	3	3
**14**	0	0	0	0	0	0
**15**	3	3	3	2	2	1
**16**	3	3	2	3	3	3
**17**	0	0	0	0	0	0
**18**	3	3	3	3	3	3
**19**	0	0	0	0	0	0
**20**	2	2	2	1	1	1
**21**	3	3	3	3	2	2
**22**	3	3	3	3	3	3
**23**	0	0	0	0	0	0
**24**	0	0	0	0	0	0
**25**	3	3	3	2	2	2
**26**	2	3	3	2	2	2
**27**	1	2	1	3	3	3
**28**	0	0	0	0	0	0

**Table 8:** Inter-rater reliability.

Rating Category	Conditional Probability	Kappa	Asymptotic Standard Error	Z	Confidence Interval	*p*-Value
0	1	1	0.049	20.494	0.904–1.000	<0.0001
1	0.267	0.21	0.049	4.309	0.114–0.306	<0.0001
2	0.35	0.197	0.049	4.039	0.101–0.293	<0.0001
3	0.7	0.515	0.049	10.562	0.419–0.611	<0.0001

**Table 9:** Intra-rater reliability in the positive cases alone.

Rating Category	Intra-Rater Reliability					
	R1	*p*-Value	R2	*p*-Value	R3	*p*-Value
1	−0.125	0.596	−0.059	0.803	−0.2	0.396
2	0.169	0.473	−0.5	0.034	−0.385	0.103
3	0.299	0.205	−0.169	0.474	−0.35	0.138

## Data Availability

The original contributions presented in this study are included in the article/Supplementary Material. Further inquiries can be directed to the corresponding author(s).

## Supplementary Materials

The following supporting information can be downloaded at: https://www.mdpi.com/article/10.3390/jcm14155319/s1, Supplementary Materials S1. Filters for preliminary research; Supplementary Materials S2. DL-SCT acquisition parameters.

## Abbreviations

The following abbreviations are used in this manuscript:

BME	Bone Marrow Edema
CaSupp	Calcium Suppression
DL-SCT	Dual-Layer Spectral CT
ED	Electron Density

## References

[1] Wong A.J.N., Wong M., Kutschera P., Lau K.K. (2021). Dual-energy CT in musculoskeletal trauma. Clin. Radiol..

[2] Delfaut E.M., Beltran J., Johnson G., Rousseau J., Marchandise X., Cotten A. (1999). Fat suppression in MR imaging: Techniques and pitfalls. RadioGraphics.

[3] Yang Y., Xu H., Xu G., Jia Z., Qian J. (2025). Dual-energy CT for evaluating bone marrow edema in rheumatoid arthritis: An observational single-center study. Rheumatology.

[4] Bernstein M.A., King K.F., Zhou X.J. (2004). Handbook of MRI Pulse Sequences.

[5] Pandey R., McNally E., Ali A., Bulstrode C. (1998). The role of MRI in the diagnosis of occult hip fractures. Injury.

[6] Popp D., Kerschbaum M., Mahr D., Thiedemann C., Ernstberger A., Wiesinger I., Bäumler W., Alt V., Schicho A. (2021). Necessity of Immediate MRI Imaging in the Acute Care of Severely Injured Patients. Medicina.

[7] McLean B., Thompson D. (2023). MRI and the Critical Care Patient: Clinical, Operational, and Financial Challenges. Crit. Care Res. Pract..

[8] Ghadimi M., Sapra A. (2022). Magnetic Resonance Imaging Contraindications.

[9] Guggenberger R., Gnannt R., Hodler J., Krauss B., Wanner G.A., Csuka E., Payne B., Frauenfelder T., Andreisek G., Alkadhi H. (2012). Diagnostic performance of dual-energy CT for the detection of traumatic bone marrow lesions in the ankle: Comparison with MR imaging. Radiology.

[10] Baffour F.I., Glazebrook K.N., Morris J.M., Michalak G.J., Fletcher J.G., Leng S., McCollough C.H. (2019). Clinical utility of virtual noncalcium dual-energy CT in imaging of the pelvis and hip. Skeletal Radiol..

[11] Hangartner T.N., Gilsanz V. (1996). Evaluation of cortical bone by computed tomography. J. Bone Miner. Res..

[12] Foti G., Serra G., Iacono V., Zorzi C. (2021). Identification of Traumatic Bone Marrow Oedema: The Pearls and Pitfalls of Dual-Energy CT (DECT). Tomography.

[13] McCollough C.H., Leng S., Yu L., Fletcher J.G. (2015). Dual- and Multi-Energy CT: Principles, Technical Approaches, and Clinical Applications. Radiology.

[14] Reagan A.C., Mallinson P.I., O’Connell T., McLaughlin P.D., Krauss B., Munk P.L., Nicolaou S., Ouellette H.A. (2014). Dual-energy computed tomographic virtual noncalcium algorithm for detection of bone marrow edema in acute fractures: Early experiences. J. Comput. Assist. Tomogr..

[15] Suh C.H., Yun S.J., Jin W., Lee S.H., Park S.Y., Ryu C.W. (2018). Diagnostic performance of dual-energy CT for the detection of bone marrow oedema: A systematic review and meta-analysis. Eur. Radiol..

[16] Pache G., Krauss B., Strohm P., Saueressig U., Blanke P., Bulla S., Schäfer O., Helwig P., Kotter E., Langer M. (2010). Dual-energy CT virtual non calcium technique: Detecting posttraumatic bone marrow lesions--feasibility study. Radiology.

[17] François M.A., Comby P.O., Goueslard K., Lebeaupin F., Lemogne B., Ricolfi F., Lenfant M. (2025). Diagnostic performance of spectral CT in detecting bone marrow edema for vertebral fracture: A multi-reader study. Eur. J. Radiol..

[18] Cao J.X., Wang Y.M., Kong X.Q., Yang C., Wang P. (2015). Good interrater reliability of a new grading system in detecting traumatic bone marrow lesions in the knee by dual energy CT virtual non-calcium images. Eur. J. Radiol..

[19] Mei K., Ehn S., Oechsner M., Kopp F.K., Pfeiffer D., Fingerle A.A., Pfeiffer F., Combs S.E., Wilkens J.J., Rummeny E.J. (2018). Dual-layer spectral computed tomography: Measuring relative electron density. Eur. Radiol. Exp..

[20] Van Abbema J.K., Van der Schaaf A., Kristanto W., Groen J.M., Greuter M.J. (2012). Feasibility and accuracy of tissue characterization with dual source computed tomography. Phys. Med..

[21] Landry G., Reniers B., Granton P.V., van Rooijen B., Beaulieu L., Wildberger J.E. (2011). Extracting atomic numbers and electron densities from a dual source dual energy CT scanner: Experiments and a simulation model. Radiother. Oncol..

[22] Altman A., Carmi R. (2009). TU-E-210A-03: A double-layer detector, dual-energy CT—Principles, advantages and applications. Med. Phys..

[23] Mallinson P.I., Coupal T.M., McLaughlin P.D., Nicolaou S., Munk P.L., Ouellette H.A. (2016). Dual-Energy CT for the Musculoskeletal System. Radiology.

[24] Petritsch B., Kosmala A., Weng A.M., Krauss B., Heidemeier A., Wagner R., Heintel T.M., Gassenmaier T., Bley T.A. (2017). Vertebral Compression Fractures: Third-Generation Dual-Energy CT for Detection of Bone Marrow Edema at Visual and Quantitative Analyses. Radiology.

[25] Kaup M., Wichmann J.L., Scholtz J.E., Beeres M., Kromen W., Albrecht M.H., Lehnert T., Boettcher M., Vogl T.J., Bauer R.W. (2016). Dual-Energy CT-based Display of Bone Marrow Edema in Osteoporotic Vertebral Compression Fractures: Impact on Diagnostic Accuracy of Radiologists with Varying Levels of Experience in Correlation to MR Imaging. Radiology.

[26] Mendonca P.R., Lamb P., Sahani D.V. (2014). A Flexible Method for Multi-Material Decomposition of Dual-Energy CT Images. IEEE Trans. Med. Imaging.

[27] Schwaiger B.J., Gersing A.S., Hammel J., Mei K., Kopp F.K., Kirschke J.S., Rummeny E.J., Wörtler K., Baum T., Noël P.B. (2018). Three-material decomposition with dual-layer spectral CT compared to MRI for the detection of bone marrow edema in patients with acute vertebral fractures. Skeletal Radiol..

[28] Mei K., Schwaiger B.J., Kopp F.K., Ehn S., Gersing A.S., Kirschke J.S., Muenzel D., Fingerle A.A., Rummeny E.J., Pfeiffer F. (2017). Bone mineral density measurements in vertebral specimens and phantoms using dual-layer spectral computed tomography. Sci. Rep..

[29] Carrafiello G., Laganà D., Pellegrino C., Mangini M., Fontana F., Piacentino F., Recaldini C., Rovera F., Dionigi G., Boni L. (2008). Ablation of painful metastatic bone tumors: A systematic review. Int. J. Surg..

[30] Fontana F., Piacentino F., Ierardi A.M., Carrafiello G., Coppola A., Muollo A., Beneventi A., Floridi C., Imperatori A.S., Carcano G. (2021). Comparison Between CBCT and Fusion PET/CT-CBCT Guidance for Lung Biopsies. Cardiovasc. Interv. Radiol..

[31] Saba L., De Filippo M., Saba F., Fellini F., Marcy P.Y., Dagan R., Voituriez P., Aelvoet J., Klotz G., Bernard R. (2019). Dual energy CT and research of the bone marrow edema: Comparison with MRI imaging. Indian J. Radiol. Imaging.

