# Supplementary material for: Dual-Layer Spectral CT with Electron Density in Bone Marrow Edema Diagnosis: A Valid Alternative to MRI?
Source: J Clin Med. 2025 Jul 28;14(15):5319. doi: 10.3390/jcm14155319 (PMC12347524; doi:10.3390/jcm14155319)
Supplement: Supplementary file 1 [file jcm-14-05319-s001.zip › jcm-3687345-supplementary.pdf]

Supplementary Materials S1. Filters for preliminary research

Supplementary Materials S1
RIS – Radiology Information System; Filters for preliminary research: “RM NH1” (i.e. MRI New Hospital 1); “RMNH2” (i.e. MRI New Hospital 2); “bone fracture”, “trauma”, “metastatic lesion”, “osteoporotic fracture”.

Supplementary Materials S2. DL-SCT acquisition parameters

Supplementary Materials S2
Tube voltage: 120 kVp; Tube current-time product: automatic tube current modulation; Gantry rotation time: 0.5 s; Detector collimation: 64*0.625 mm; Z-coverage: 40 mm; Slice thickness/gap: 1.0 / 0.0 mm; Pitch: 0.801; Matrix size: 512x512; Field of view: patient-dependent; Reconstruction kernel: Standard (B); Iterative reconstruction: iDose4 - Level 4).
